# Coordinated Approaches to Strengthen State and Local Public Health Actions to Prevent Obesity, Diabetes, and Heart Disease and Stroke

**DOI:** 10.5888/pcd15.170493

**Published:** 2018-01-25

**Authors:** Gia E. Rutledge, Kimberly Lane, Caitlin Merlo, Joanna Elmi

**Affiliations:** 1 Division of Diabetes Translation, National Center for Chronic Disease Prevention and Health Promotion, Centers for Disease Control and Prevention, Atlanta, Georgia; 2 Division of Nutrition, Physical Activity, and Obesity, National Center for Chronic Disease Prevention and Health Promotion, Centers for Disease Control and Prevention, Atlanta, Georgia; 3 Division of Population Health, National Center for Chronic Disease Prevention and Health Promotion, Centers for Disease Control and Prevention, Atlanta, Georgia; 4 Division for Heart Disease and Stroke Prevention, National Center for Chronic Disease Prevention and Health Promotion, Centers for Disease Control and Prevention, Atlanta, Georgia

Chronic diseases, including heart disease, stroke, cancer, diabetes, and obesity, are the leading causes of death in the United States and account for most of the nation’s health care costs (1). Heart disease is the leading cause of death among men and women in the United States, accounting for 1 of every 4 deaths (1). Approximately 140,000 Americans die each year from stroke, and it is a leading cause of long-term disability (2,3). It is estimated that more than 9% of the US population has diabetes, which is the leading cause of kidney failure, lower-limb amputations other than those caused by injury, and new cases of blindness among adults (4). Additionally, more than one-third of US adults have obesity, which is associated with several chronic conditions (5,6).

Chronic diseases are common and costly, but many are preventable. Although it is important to address the underlying risk factors for chronic diseases at the individual level, it is also critical to implement population-based interventions, including health-promoting policies and environments that affect where we work, live, play, and receive health care. This requires a multifaceted approach and the collective efforts of federal, state, local, private, and community-based organizations along with national partners.

The Centers for Disease Control and Prevention’s (CDC’s) mission is to prevent or control all diseases that affect Americans (7). CDC puts science into action by tracking diseases and determining their causes and by identifying the most effective ways to prevent and control them (7). This work entails tackling the major health problems that cause death and disability for Americans and promoting healthy and safe behaviors, communities, and environments (7). 

The mission of CDC’s National Center for Chronic Disease Prevention and Health Promotion (NCCDPHP) is to “help people and communities prevent chronic diseases and promote health and wellness for all” (8). NCCDPHP supports disease control efforts through 5-year term funding mechanisms called cooperative agreements that are awarded to state and local public health agencies to strengthen partnerships to improve health at the community level (9). In 2013, NCCDPHP developed the State Public Health Actions to Prevent Obesity, Diabetes, and Heart Disease and Stroke (State Public Health Actions [SPHA]-1305), a cooperative agreement that combined the efforts of 4 CDC divisions: the Division for Heart Disease and Stroke Prevention (DHDSP); the Division of Diabetes Translation (DDT); the Division of Nutrition, Physical Activity, and Obesity (DNPAO); and the Division of Population Health’s School Health Branch (SHB). The agreement funded 50 state health departments and the District of Columbia to implement strategies in health systems and communities to prevent chronic disease and reduce complications associated with them (10). State Public Health Actions provides examples of how mutually reinforcing strategies are implemented. Two tiers of strategies were recommended, basic and enhanced (Box 1).

**Box 1. Strategies of State Public Health Actions to Prevent and Control Diabetes, Heart Disease, Obesity, and Associated Risk Factors and Promote School Health (SPHA-1305)**

**Table:** 

**BASIC STRATEGIES**
Promote the adoption of food service guidelines/nutrition standards, which include sodium
Promote the adoption of physical education/physical activity in schools
Promote adoption of physical activity in early care and education and worksites
Promote reporting of blood pressure and hemoglobin A1c measures; and as able, initiate activities that promote clinical innovations, team-based care, and self-monitoring of blood pressure
Promote awareness of high blood pressure among patients
Promote awareness of prediabetes among people at high risk for type 2 diabetes
Promote participation in diabetes self-management education programs
**ENHANCED STRATEGIES**
**Environmental approaches to promote health and support and reinforce healthful behaviors**
Access to healthy food and beverages
Food service guidelines/nutrition standards where foods and beverages are available. Guidelines and standards should address sodium
Supportive nutrition environments in schools
Physical activity access and outreach
Physical activity in early childhood education
Quality physical education and physical activity in kindergarten through 12th grade in schools
Access to breastfeeding-friendly environments
**Health system interventions to improve the effective delivery and use of clinical and other preventive services**
Quality improvement processes in health systems
Use of team-based care in health systems
**Community–clinical linkages to support cardiovascular disease and diabetes prevention and control efforts**
Use of diabetes self-management education programs in community settings
Use of CDC-recognized lifestyle intervention programs in community settings for the primary prevention of type 2 diabetes
Use of health-care extenders in the community in support of self-management of high blood pressure and diabetes
Use of chronic disease self-management programs in community settings
Placement of policies, processes, and protocols in schools to meet the management care needs of students with chronic conditions

Each of the 4 divisions focuses on a specific area of chronic disease. DHDSP provides public health leadership to improve cardiovascular health for all Americans and to reduce the burden and end disparities related to heart disease and stroke (www.cdc.gov/dhdsp/index.htm). DDT supports programs and activities to prevent or delay the onset of type 2 diabetes and to improve health outcomes for people diagnosed with diabetes (www.cdc.gov/diabetes/home/index.html). DNPAO focuses on decreasing obesity in the United States by encouraging regular physical activity and good nutrition at every stage of life. DNPAO supports healthy eating, active living, and obesity prevention by creating healthy child care centers, hospitals, schools, and worksites; building the capacity of state health departments and national organizations; and conducting research, surveillance, and evaluation studies (www.cdc.gov/nccdphp/dnpao/index.html). SHB’s aims are to improve the well-being of youth through healthy eating, physical education, and physical activity; to reduce risk factors associated with childhood obesity; and to manage chronic health conditions in schools (www.cdc.gov/healthyschools/stateprograms.htm).

The primary purpose of SPHA-1305 is to support state-level and statewide implementation of cross-cutting, evidence-based strategies to promote health and prevent and control chronic diseases and their risk factors (11). SPHA-1305 uses a collective approach to 1) improve environments in worksites, schools, early childhood education services, state and local government agencies, and community settings to promote healthy behaviors and expand access to healthy choices for people of all ages related to diabetes, cardiovascular health, physical activity, healthy foods and beverages, obesity, and breastfeeding; 2) improve the delivery and use of quality clinical and other health services aimed at preventing and managing high blood pressure and diabetes; and 3) increase links between community and clinical organizations to support prevention, self-management, and control of diabetes, high blood pressure, and obesity (10). The ultimate goal of SPHA-1305 is to make healthy living easier for all Americans. The following are primary outcomes of SPHA-1305:

- Increased consumption of a healthy diet
- Increased physical activity across the life span
- Improved medication adherence for adults with high blood pressure or diabetes
- Increased self-monitoring of high blood pressure tied to clinical support
- Increased access to and participation in diabetes self-management programs and type 2 diabetes prevention programs
- Increased breastfeeding

In 2014, CDC developed a second cooperative agreement, State and Local Public Health Actions to Prevent Obesity, Diabetes, and Heart Disease and Stroke (SLPHA-1422), a program designed for states and large cities to implement strategies to control and prevent chronic disease through a dual approach — targeting both the overall population and priority populations (groups of people who are at high risk of chronic disease, are experiencing a disproportionate incidence of chronic diseases and conditions, or are experiencing racial/ethnic or socioeconomic disparities). This competitive cooperative agreement combined the efforts of 3 NCCDPHP divisions (DDT, DNPAO, and DHDSP), and was awarded to 17 states and 4 large cities to implement additional evidence-based strategies to expand the reach and impact of SPHA-1305 with the aim of reducing health disparities and improving health equity among adults. SLPHA-1422 supports interventions to prevent obesity, type 2 diabetes, heart disease, and stroke (through control of high blood pressure) and to reduce health disparities in the prevalence of these among adults in the population overall and in priority populations (12). SLPHA-1422 awardees used the dual approach and mutually reinforcing strategies to maximize the impact of strategies implemented in SPHA-1305 by working with partners and funding subawardees at the local level. By applying the dual approach, states and large cities implemented strategies to improve the health of the whole population and of priority populations (12). The strategies are described as mutually reinforcing because they are implemented simultaneously and synergistically to address multiple risk factors and chronic diseases (12). 

Three tiers of strategies make up SLPHA-1422, environmental strategies, health system strategies, and community–clinical linkage strategies. The purpose of SPHA-1422 environmental strategies is to “support environmental and system approaches to promote health, support and reinforce healthful behaviors, and build support for lifestyle improvements for the general population and particularly for those with uncontrolled high blood pressure and those at high risk for developing type 2 diabetes” (12). The purpose of community–clinical linkage strategies is to “support health system interventions and community–clinical linkages that focus on the general population and priority populations” (Box 2) (12). Environmental strategies were implemented in the same communities and jurisdictions as health system strategies and community–clinical linkage strategies, with local improvements supported by statewide efforts funded by this cooperative agreement as well as those supported by SPHA-1305. The following are primary outcomes of SLPHA-1422:

- Increased consumption of nutritious food and beverages and increased physical activity
- Increased engagement in lifestyle change to prevent type 2 diabetes
- Improved medication adherence for adults with high blood pressure
- Increased self-monitoring of high blood pressure tied to clinical support
- Increased referrals to and enrollment in CDC-recognized lifestyle change programs to prevent type 2 diabetes

**Box 2. State and Local Public Health Actions to Prevent Obesity, Diabetes, and Heart Disease and Stroke (SLPHA-1422) Strategies**

**Table:** 

**COMPONENT 1**
**Environmental strategies to promote health and support and reinforce healthful behaviors**
Implement food and beverage guidelines including sodium standards (ie, food service guidelines for cafeterias and vending machines) in public institutions, worksites, and other key locations, such as hospitals
Strengthen access to and sales of healthy foods (eg, fruit and vegetables, more low/no sodium options) in retail venues (eg, grocery stores, supermarkets, chain restaurants, markets) and community venues (eg, food banks) through increased availability and improved pricing, placement, and promotion
Strengthen community promotion of physical activity though signage, worksite policies, social support, and joint-use agreements
Develop and/or implement transportation and community plans that promote walking
**Strategies to build support for lifestyle change, particularly for those at high risk, to support diabetes, heart disease, and stroke prevention efforts**
Plan and execute strategic data-driven actions through a network of partners and local organizations to build support for lifestyle change. Implement evidence-based engagement strategies (eg, tailored communications, incentives) to build support for lifestyle change
Increase coverage for evidence-based supports for lifestyle change by working with network partners
**COMPONENT 2**
**Health system interventions to improve the quality of health care delivery to populations with the highest hypertension and prediabetes disparities**
Increase the adoption of electronic health records and the use of health information technology to improve performance (eg, implement advanced Meaningful Use data strategies to identify patient populations who experience cardiovascular disease–related disparities)
Increase the institutionalization and monitoring of aggregated/standardized quality measures at the provider level (eg, use dashboard measures to monitor health care disparities, implement activities to eliminate health care disparities)
Increase engagement of nonphysician team members (ie, nurses, pharmacists, dietitians, physical therapists and patient navigators/community health workers) in hypertension management in community health care systems
Increase use of self-measured blood pressure monitoring tied with clinical support
Implement systems to facilitate identification of patients with undiagnosed hypertension and people with prediabetes
**Community–clinical linkage strategies to support heart disease, stroke, and type 2 diabetes prevention efforts**
Increase engagement of community health workers to promote linkages between health systems and community resources for adults with high blood pressure and adults with prediabetes or at high risk for type 2 diabetes
Increase engagement of community pharmacists in the provision of medication self-management for adults with high blood pressure
Implement systems to facilitate bi-directional referral between community resources and health systems, including lifestyle change programs (eg, electronic health records, 800 numbers, 211 referral systems)

This special collection of articles in *Preventing Chronic Disease* describes how SPHA-1305 and SLPHA-1422 use a coordinated approach to chronic disease prevention and control. The collection describes an evaluation approach that was designed for state and local health departments with differing levels of evaluation capacity and highlights early outcomes at the national, state, and local levels. This special collection contains 12 articles: 4 by state health departments, 2 by one large city, and 6 authored by CDC staff members. Articles highlight a range of SPHA-1305 and SLPHA-1422 strategies. An article by Park et al describes in detail the foundations for SPHA-1305, the strategies recommended by each NCCDPHP division, the administrative and management structure, and the model for providing cross-division program and evaluation technical assistance (13). Given this complex approach to implementing a national chronic disease prevention initiative, it was imperative that the evaluation design use a robust, multi-tiered approach to accountability and learning. This comprehensive evaluation approach is described by Vaughan et al (14).

Smith et al summarize Maryland’s approach to improving implementation of quality improvement processes in Federally Qualified Health Centers through the use of health information technology and standardized reporting of clinical quality measures (15). Other states interested in learning how to harness the potential of electronic health records and how to use population health data to drive improvements in quality of care will appreciate this step-by-step explanation of how to gain the buy-in of health centers and how to build the operational structure of a data warehouse. The article also discusses challenges encountered in the process and plans for scaling up these efforts.

Oser et al describe how the Montana Department of Public Health and Human Services used SPHA-1305 funding to conduct an evaluation of a 3-year intervention among 25 community pharmacies in rural areas to improve adherence to blood pressure medication (16). In addition to patient-level data, Montana also implemented a statewide survey of pharmacists and identified barriers perceived from the pharmacy point of view. Results indicate that the intervention was successful with promising improvements in patient medication adherence.

Barragan et al focus on pharmacy-led strategies that the Los Angeles County Department of Public Health implemented with SLPHA-1422 funding (17). Authors report results from a community and stakeholder needs assessment for pharmacist services for management of hypertension medication therapy. The needs assessment included 3 components: 1) a policy context scan, 2) a survey of participants in a pharmacy leadership symposium, and 3) an internet public opinion survey of a final sample of more than 1,000 English- and Spanish-speaking Los Angeles County residents. A synthesis of results from these 3 assessments produced a list of needs and assets for scaling up and spreading pharmacy-led patient care services in Los Angeles County.

Mosst et al describe a practice-grounded framework used by the Los Angeles County Health Department to scale and sustain the National Diabetes Prevention Program (National DPP) by using a diverse partner network (18). By developing a 3-pronged framework (expanding outreach and education, improving health care referral systems and protocols, and increasing access to insurance coverage for the National DPP), Los Angeles County took an approach that other large jurisdictions can use to identify people with prediabetes and expand access to and use of CDC-recognized type 2 diabetes prevention programs.

Mensa-Wilmot et al use a mixed-method evaluation approach to describe preliminary findings of a collaborative effort between CDC and state health departments designed to scale and sustain the National DPP (19). Grantees reported reimbursement availability, practice and provider referral policies, and having standard curricula as facilitators to implementing the National DPP lifestyle change program. Understanding activities implemented by grantees and the barriers and facilitators they identify is critical for developing relevant and timely technical assistance and for understanding the impact of the program.

Morgan et al describe activities state health departments implemented to increase referrals to, coverage for, and availability of diabetes self-management education and support (DSMES) programs (20). By year 3 of SPHA-1305, more than 3,000 DSME programs had been established in 41 states. State health departments contributed to these increases by assisting organizations in establishing new DSME programs, providing technical assistance to providers, convening stakeholders to address gaps in DSME insurance coverage, and using marketing strategies to educate patients about the importance of DSME. Conducting early assessments of the activities implemented by state health departments and analyzing progress in performance measures associated with them provides early outcome results that can be used to develop technical assistance to help grantees identify where more focus is needed to further improve results by the end of the 5-year cooperative agreement.

An article by Fritz et al examines the SPHA-1305 strategy of increasing physical activity through community design (21). In this community case study, the authors describe how the Indiana State Department of Health used a workshop model to support communities with implementation of active-living opportunities in their communities to improve or increase access to physical activity. The authors report that providing a workshop model with follow-up support to the community resulted in policy adoption, the creation of new advisory committees, and new local funding allocations for active-living projects. These findings may inform efforts of other state health agencies as they collaborate with communities to improve physical access.

Geary et al describe the extent to which 38 states’ Quality Rating and Improvement Systems (QRIS) include obesity prevention content (22). States can use QRIS to set standards that define high-quality care and to award child care programs with a quality rating designation based on how well they meet these standards (eg, a star rating). The authors reviewed each state’s QRIS standards and compared them with the 47 “high impact” obesity prevention standards contained in *Caring for Our Children: National Health and Safety Performance Standards; Guidelines for Early Care and Education Programs, 3rd Ed (Caring for Our Children)* (23). The authors found that of 38 states with publically available standards, 20 included at least one standard with obesity prevention content; however, most had fewer than 5, suggesting room for states to embed additional obesity prevention standards into QRIS.

The article by Papa et al examines 5 of the child care standards of the Arizona Department of Health Services related to obesity prevention that are part of the Arizona Empower Program, a program that promotes healthy environments for children in Arizona’s licensed child care facilities (24). The authors examined 2 years of statewide data, tracked progress in implementing these 5 Empower standards, and identified areas in which facilities needed additional support to fully implement the standards. The results indicate that 1 in 5 facilities fully implemented all 5 standards, with the staff training standard having the highest level of implementation across facilities (77%) and the breastfeeding standard having the lowest implementation (44%). These findings can inform training and technical assistance efforts to further support the implementation of these standards in Arizona’s licensed child care facilities.

An article by Pitt Barnes et al examines performance measures and reported evaluation data from all 51 awardees to assess progress in improving the school nutrition environment and services over the first 4 years of the program (24). Findings indicated that, compared with year 2, by year 4 awardees made significant progress, especially related to providing professional development on strategies to improve the school nutrition environment, adopting and implementing policies to establish standards (including standards for sodium) for all competitive foods available during the school day, not selling unhealthy foods and beverages during the school day, placing fruits and vegetables near the cafeteria cashier where they are easy to access, and providing information to students or families on the nutrition, calorie, and sodium content of foods available. However, the data also show that only 33.5% of local education agencies adopted and implemented policies that prohibit all forms of advertising and promotion of unhealthy foods and beverages. Because the federal requirement for local school wellness policies now includes addressing the marketing of unhealthy foods, additional training, technical assistance, and guidance is likely needed to help districts adopt marketing policies.

This special collection describes overarching approaches and examples of interventions implemented by state and local health departments to prevent and manage obesity, diabetes, heart disease, and stroke. Readers should note that these articles represent early evaluation results of both SPHA-1305 and SLPHA-1422 and demonstrate promise that the implemented strategies are reaching populations in need and are beginning to have a population-wide impact. As of 2016, the 2 national programs are in the final year of funding. With ongoing analysis of performance-measure data, the impact of these programs will continue to be examined and reported.

Collectively, the work of SPHA-1305 and SLPHA-1422 demonstrates the barriers and facilitators that affect state and local program development, implementation, and evaluation of chronic disease prevention initiatives and describes a coordinated approach to implementing programs. This information will inform other state and local programs and further the potential reach of these approaches. The findings presented in this special collection contribute practice-based knowledge to the field of chronic disease prevention and management, evidence of combining different disease-specific funding streams to achieve early outcomes with greater efficiency, and lessons learned for future coordinated national chronic disease programs.
